# Adult-onset hereditary myeloid malignancy and allogeneic stem cell transplantation

**DOI:** 10.3389/fonc.2022.997530

**Published:** 2022-09-16

**Authors:** Takashi Toya, Hironori Harada, Yuka Harada, Noriko Doki

**Affiliations:** ^1^ Hematology Division, Tokyo Metropolitan Cancer and Infectious Diseases Center, Komagome Hospital, Tokyo, Japan; ^2^ Laboratory of Oncology, School of Life Sciences, Tokyo University of Pharmacy & Life Sciences, Tokyo, Japan; ^3^ Clinical Research Support Center, Tokyo Metropolitan Cancer and Infectious Diseases Center, Komagome Hospital, Tokyo, Japan

**Keywords:** hereditary myeloid malignancy, germline mutation, genetic testing, allogeneic stem cell transplantation, donor cell leukemia

## Abstract

Hereditary myeloid malignancies, especially in adults or elderly persons, had been considered quite rare before the next-generation sequencing era; however, increased usage of clinical sequencing has revealed much higher prevalence of inherited myeloid malignancies. *DDX41* and various pathogenic germline mutations have newly been recognized as the cause of adult-onset familial leukemia and myeloid malignancies. Although germline predisposition to myeloid neoplasms had been categorized as a provisional entity in the World Health Organization classification of hematopoietic neoplasms in 2016, methodology for the identification of hereditary myeloid malignancies has not been fully established yet. In addition, many unresolved problems, such as epidemiology, the exact pathogenic mechanisms, and ideal treatment strategy, including indications of allogeneic hematopoietic stem cell transplantation, still remain. Related donor selection for stem cell transplant is a particularly sensitive issue due to the possibility of germline mutation of the candidate relatives and the risk of donor cell leukemia after transplantation. Here, we reviewed the current evidence regarding epidemiology, diagnosis, mechanisms of progression, and transplantation strategy for hereditary myeloid malignancies.

## Introduction

Hereditary myeloid malignancies (HMMs) are myeloid neoplasms that arise in individuals with germline mutations associated with increased risk of myeloid malignancies. The first report of HMMs were published in 1861, although the causality remained unclear (1); HMMs had been considered an extremely rare disease for a long time. In 1999, germline *RUNX1* mutation was identified, for the first time, as the genetic background of familial platelet disorder with predisposition to myeloid malignancy (FPD-MM). Subsequent advent of next-generation sequencing (NGS) era unraveled HMMs as much more manifold and common diseases than considered earlier (2–6). In 2016, HMMs were defined as “myeloid neoplasms with germline predisposition” in the revised fourth edition of the World Health Organization (WHO) classification of myeloid neoplasms (7), and were renamed as “myeloid neoplasms associated with germline predisposition” in the fifth edition of WHO classification (8). Candidate genes associated with HMMs, such as *GATA2*, *CEBPA*, *ETV6*, *ANKRD26*, *SAMD9*, and *SAMD9L*, have been discovered subsequently (9). Recent reports have shown that approximately 5–10% of patients with hematological malignancy carry a germline variant (10). However, the penetrance is not 100% in HMMs, since not all patients with a germline variant develop hematological malignancy. Moreover, *de novo* germline variant may occur where an obvious family history could be lacking. Some cases with HMMs are often elderly-onset, especially in individuals with germline *DDX41* mutations (11). Therefore, the accurate and prompt diagnosis of HMMs is often challenging.

Allogeneic hematopoietic stem cell transplantation (HSCT) is the only curative intervention for HMMs. However, the optimal strategy for HSCT, including indication, timing, donor selection, conditioning regimen, and toxicity management has not been fully elucidated yet. Although many guidelines recommend HSCT for HMMs, data regarding transplant outcome are highly limited (12). In this manuscript, we reviewed the pathophysiology, clinical characteristics, and recent evidence regarding HSCT for HMMs. The review focused on adult-onset HMMs, which often get involved with solitary adult myeloid malignancies in clinical practice. Some child-onset hereditary disorders are beyond the scope of the current review, and the relevant guidelines should be perused in that regard.

## Clinical features corresponding to each mutation

In general, HMMs are classified into three groups (**Table 1**), namely (1) myeloid neoplasms with germline predisposition and pre-existing platelet disorder (*RUNX1*, *ANKRD26*, and *ETV6*), (2) myeloid neoplasms with germline predisposition and potential organ dysfunction (*GATA2*, *SAMD9*, *SAMD9L*, etc.), and (3) myeloid neoplasms with germline predisposition without a preexisting platelet disorder or organ dysfunction (*CEBPA* and *DDX41*). Four representative genes responsible for adult-onset HMMs, namely *RUNX1*, *GATA2*, *CEBPA*, and *DDX41*, are described in detail below.

**Table 1:** Major subtypes of myeloid neoplasms associated with germline predisposition^8^.

Germline variant	Major hematologic disorder	Other characteristics
**Myeloid neoplasms with germline predisposition without a preexisting platelet disorder or organ dysfunction**		
*CEBPA* ^13, 14^	AML	None
*DDX41* ^15-17^	MDS, AML	None
*TP53* ^18, 19^	ALL, myeloid neoplasms	Cancer predisposition
**Myeloid neoplasms with germline predisposition and pre-existing platelet disorder**		
*RUNX1* ^13, 20^	MDS, AML	Thrombocytopenia, decreased platelet function
*ANKRD26* ^9, 21^	AML, MDS, CML	Thrombocytopenia, decreased platelet function
*ETV6* ^18, 21^	ALL, MDS, AML	Thrombocytopenia, decreased platelet function
**Myeloid neoplasms with germline predisposition and potential organ dysfunction**		
*GATA2* ^13, 22^	MDS, AML, BMF	Monocytopenia, B lymphocytopenia, lymphoedema, pulmonary alveolar proteinosis, congenital deafness
*SAMD9* ^18, 23^	MDS, AML, BMF	Infection, growth restriction, adrenal hypoplasia, genital phenotypes, enteropathy
*SAMD9L* ^18^	MDS, AML, BMF	Ataxia, systemic autoinflammatory diseases

ALL, acute lymphoblastic leukemia; AML, acute myeloid leukemia; BMF, bone marrow failure; CML, chronic myeloid leukemia; MDS, myelodysplastic syndromes.

### RUNX1

RUNX1 is a critical transcription factor for hematopoiesis; *RUNX1* mutation is recurrently detected and is a poor prognostic marker in myeloid malignancies (24). FPD-MM is the first HMMs whose genetic causality was recognized in 1999 (20). A germline *RUNX1* mutation is associated with predisposition to mainly acute myeloid leukemia (AML) and myelodysplastic syndromes (MDS) while some cases with various lymphoid malignancies have also been reported (25–28). FPD-MM accounts for 8–30% of AML cases with *RUNX1* mutations (29–33).

Chronic thrombocytopenia and/or bleeding tendency is a characteristic of FPD-MM pedigree. Some pedigrees may be considered as immune thrombocytopenic purpura. Approximately 40% of the family members with blood relationship develop myeloid malignancy during their lifetime (34), although significant heterogeneity is evident across families (35). Churpek et al. had reported that cumulative risk of developing clonal hematopoiesis by 50 years of age is > 80% (36). Heterozygous *RUNX1* germline mutation alone is not sufficient for leukemogenesis in this context, and additionally acquired mutations, such as gene mutations in *ASXL1*, *FLT3*, *WT1* and *CDC25C*, may also be important for malignant transformation (37–39).

### GATA2

MonoMAC syndrome is characterized by monocytopenia, B lymphocytopenia, pulmonary alveolar proteinosis (PAP), and frequent *M. avium* complex infection. Patients with Emberger syndrome exhibit primary lymphoedema, cutaneous warts, and sensorineural deafness. Dendritic cell, monocyte, and B- and NK-lymphoid deficiencies occur with DCML deficiency. The patients frequently develop familial MDS/AML, although the pathogenesis remains to be clarified. In 2011, Hahn et al. revealed that loss-of-function germline GATA-binding protein 2 (*GATA2*) mutations are associated with all the above-mentioned syndromes (22). Currently, the syndromes are collectively called *GATA2* deficiency. Opportunistic viral and mycobacterial infections are frequently observed in patients with *GATA2* deficiency. Interestingly, interindividual variation of clinical phenotypes is significant even among patients with the same germline mutation (21).

*GATA2* deficiency is the most common cause of childhood MDS, and monosomy 7 and *ASXL1* mutation are often accompanied by the development of MDS and AML in cases with germline *GATA2* mutation (40). Donadieu et al. analyzed 79 patients with *GATA2* deficiency and reported 92% of them to develop some symptom at 40 years of age (41).

### CEBPA

Germline mutations in CCAAT enhancer binding protein alpha (*CEBPA*) that predispose an individual to AML were first reported in 2004 (42). Among the patients with AML having *CEBPA* mutations, approximately 7–11% had those of germline origin (43, 44). Individuals with a germline *CEBPA* mutation do not have specific phenotype before leukemia onset. In general, the onset age of leukemic progression in patients with germline *CEBPA* mutations is lower than that in patients with germline *RUNX1* and *GATA2* mutations (13). Importantly, *CEBPA* germline mutations are autosomal dominant inheritance and the penetrance of AML is reported to be nearly 100% (14).

Most patients with familial AML having germline *CEBPA* mutations have been reported to possess both the N-terminal germline mutation and the C-terminal acquired mutation (14, 45). N-terminal mutations are known to generate p30 isoforms, which have dominant negative effect (46). Most sporadic AML cases with *CEBPA* mutations were reported to possess C-terminal mutations or both (14), suggesting the importance of secondary C-terminal somatic mutations.

### DDX41

Germline mutations in DEAD/H-box helicase gene (*DDX41*) were reported for the first time in 2015 (15). Despite being a recent discovery, *DDX41* mutation is the most common genetic predisposition to MDS/AML, representing 1–5% of myeloid malignancies (11, 16, 17). Many HMMs cases with germline *DDX41* mutations were accompanied by somatic *DDX41* mutations (16, 47). In addition, remarkable ethnic deviation was found in the mutation sites in *DDX41*; however, second hit was predominantly R525H, irrespective of ethnicity (47–49). *TP53* and *ASXL1* somatic mutations are recurrently detected (48), although the prognostic impact of concomitant mutations is limited (50).

AML with germline *DDX41* mutations has unique clinical characteristics, such as approximately 3:1 male predominance, frequent absence of family history, and indolent clinical course (18, 50, 51). Makishima et al. reported that the age of progression in individuals with germline *DDX41* mutation was solely greater than 40 years, and penetrance of pathogenic *DDX41* germline variants was estimated to be 38.5% at the age of 85. In addition, they also suggested outstanding efficacy of hypomethylating agents in patients with HMMs having *DDX41* mutations (52).

## Diagnosis

Considering the low prevalence of hematological neoplasms, family history of hematological disorder is a key circumstantial evidence of HMMs, and mutation analysis should be done; however, it should be noted that lack of family history cannot deny the possibility of HMMs completely (53). Patients with signs/symptoms indicative of HMMs and those younger than 50 years should undergo genetic testing (12). However, considering the frequent absence of family history and the elderly onset in patients with germline *DDX41* mutations (51), genetic tests can be also applied for patients older than 50 years old.

Genetic testing of non-hematological tissue is the standard option to confirm the diagnosis of HMMs, and the most authorized method is a culture of fibroblasts obtained from a skin biopsy (54, 55). Although the technique can avoid contamination of blood cells, it is complicated, and buccal swab, nails, or hair roots are often used as alternatives (21).

In addition, if NGS panel in clinical sequencing detects a suspected mutation with variant allele frequency close to 50 or 100%, further testing should be done. Mutations remaining in hematologic complete remission (CR) also suggested the possibility of germline origin (37), although clonal hematopoiesis (CH) can as well persist (56, 57). The NGS panel approaches are able to efficiently identify single nucleotide variants and small insertion/deletion in target region, but can overlook copy number variants or loss of heterozygosity (58–60). Especially when the patient could be a candidate for HSCT, early diagnosis of HMMs is important, since the diagnosis can affect donor selection, as described below.

## First-line treatment for patients with myeloid malignancies

There is mostly no evidence about first-line therapy about HMMs and the optimal treatment strategies for HMMs have not been sufficiently established. However, in general, patients with HMMs, who developed myeloid malignancies, are treated as the patients with sporadic myeloid malignancies, i.e., intensive chemotherapy for fit patients with AML and using demethylating agents for patients with MDS (21). Targeted therapy can also be administered when adequate molecular target is available, although specific data for HMMs are not yet available. Whether chemosensitivity in patients with HMMs is different from that in sporadic cases is unclear, except that CR rate of patients with AML having germline *DDX41* mutations after induction chemotherapy was higher than that of patients with wild-type *DDX41* (94% vs. 69%) (16). More vigorous studies to explore the suitable initial treatment strategies are warranted.

## Indication and timing of transplantation

The suitable indication and timing of HSCT is still unclear. However, in general, long-term remission can only be obtained by allogeneic HSCT, and HSCT should be considered soon after diagnosis of myeloid malignancy, except for cases with germline *CEBPA* mutations (61).

For patients with AML having a germline *CEBPA* mutation, HSCT in CR1 is not routinely recommended because of modest survival outcome after chemotherapy and the risk of morbidity and mortality after HSCT (12, 21, 45). However, the appropriateness of refraining from HSCT in CR1 has not been sufficiently validated yet, because prospective and/or controlled studies are still lacking due to the rarity of the disease. Considering the high relapse rate and/or secondary leukemia development after chemotherapy (14), availability of a suitable donor, low dose intensity/density of chemotherapy due to side effects, and development of safer HSCT strategy may rationalize HSCT in CR1. In addition, prognostic impact of the mutation site has been reported in sporadic AML with *CEBPA* mutation (62, 63). Clinical consequences of *CEBPA* mutation site in AML with germline mutation should be clarified, although it is difficult due to the remarkable rarity of C-terminal *CEBPA* germline mutation.

Prophylactic HSCT before transformation is another strategy, but it is not commonly performed due to the risk of morbidity and mortality. However, different from other HMMs, *GATA2* deficiency can be treated with HSCT before transformation. Although optimum timing and indication of HSCT in *GATA2* deficiency is still unclear, frequent/severe infection and serious organ damage, such as pulmonary function, can be a trigger for launching HSCT (64). Norwegian nationwide survey revealed that approximately 80% of patients with symptomatic *GATA2* deficiency need HSCT (65).

In cases with a germline variant but without cytopenia and/or dysplasia, bone marrow should be assessed at the time of diagnosis, and be followed every six months or every year (53).

## Donor selection and risk of donor cell leukemia

HSCT from a donor with predisposition to myeloid malignancy should be withheld due to the risk of donor cell leukemia (DCL) (66–68). DCL is a rare post-HSCT complication and cumulative incidence has been estimated to be approximately 0.16-0.70% 15 years after HSCT (69, 70). The exact incidence of DCL in HMMs setting is unknown, but Williams et al. reviewed 19 DCL cases with genetic sequencing results and at least three of them had germline predisposing mutations (71); it suggested significant proportion of DCLs to have been derived from donors with germline mutation.

Related donor candidates of patients with HMMs should undergo genetic testing as well as HLA typing; importantly, genetic counseling before testing is also essential (12). When only a matched related donor with predisposing germline mutation is available and the relapse risk without HSCT is very high, there is no consensus about necessary and sufficient condition of the donor because the quantitative risk of DCL has not been clarified.

Genetic testing of a donor might add fuel to the problems. Gibson et al. had shown donor CH to be associated with improved recipient survival due to reduced relapse risk (70). They also showed donor *DNMT3A*-CH to be associated with lower relapse risk and superior survival only when post-transplant cyclophosphamide (PT-CY) GVHD prophylaxis was not used. The situation is more complicated, since Crysandt et al. suggested germline predisposition to be extended to polymorphisms (21). Inamoto et al. had reported the association between donor/recipient polymorphisms and the risk of sclerotic GVHD after HSCT (72). Should a donor candidate test HLA, germline predisposition, CH, and polymorphisms? The obligation of the bona fide donor is large, and exploration of the “perfect” donor can reduce donor availability. Suitable biomarkers for quantitative evaluation of the relevant risks are highly required.

Among alternative donors, comparison between umbilical cord blood (UCB) and haploidentical donor remains a matter of debate (73). In most patients with HMMs, little evidence exists about alternative donor selection. However, in patients with *GATA2* deficiency, Grossman et al. reported poor outcome of UCB transplantation (73) and Nichols-Vinueza et al. reported excellent outcome of haploidentical transplantation with PT-CY GVHD prophylaxis (74). At least in HSCT for *GATA2* deficiency, haploidentical transplantation with PT-CY may be more suitable compared with UCB transplant.

## Conditioning regimen

There is no recommended conditioning regimen yet specific for patients with HMMs. In general, adult patients with HMMs are younger than patients with sporadic myeloid malignancies, and myeloablative conditioning can be applied in more cases than in sporadic cases. However, reduced intensity conditioning is preferred in patients with *GATA2* deficiency due to comorbidities such as concomitant infections and PAP (73).

High-dose total body irradiation (TBI)-regimens are avoided in cases with germline DNA damage response gene mutations due to the risk of second cancer after HSCT. For example, in patients with Fanconi anemia, non-TBI- or low dose-TBI (4 Gy)-containing regimens are usually employed (75). Although some categories of HMMs, such as Fanconi anemia and Li-Fraumeni syndrome, indicate high-risk feature (19, 76), the risk of second cancer after HSCT in most patients with HMMs is unclear, and further accumulation of cases would be necessary to answer these questions.

## Complications after transplantation

In patients with myeloid neoplasms and germline predisposition without potential organ dysfunction (i.e., patients with germline mutation in *RUNX1*, *DDX41*, *CEBPA* and so on), no specific complication after HSCT has been reported till date. However, there is no prospective as well as large-scale retrospective study which focus on transplant complications in patients with HMMs; therefore, we have limited data about post-HSCT complications in HMMs and future studies in these settings are necessary.

Adult patients with germline predisposition and potential organ dysfunction practically refer to patients with *GATA2* deficiency, since nearly all patients with *SAMD9*/*SAMD9L* germline mutations, i.e. MIRAGE syndrome and Ataxia pancytopenia syndrome, are diagnosed and treated in their childhood (23). For HSCT in patients with *GATA2* deficiency, variable comorbidities could complicate the transplantation procedures. For example, many patients with *GATA2* deficiency suffer from nontuberculous mycobacteria (NTM) and human papillomavirus (HPV) infections in their clinical courses. However, previous reports had suggested that reactivation of NTM during HSCT could be quite rare under appropriate antimycobacterial therapy up to at least three months after transplantation (73). Drug interaction between calcineurin inhibitors (CIs) and anti-NTM drugs might affect the concentration of CIs (77), and hepatic toxicity due to anti-NTM drugs should also be noted. Reactivation of HPV after HSCT seems to be also rare (73). Patients with *GATA2* deficiency frequently develop PAP, which can cause severe respiratory failure. Early HSCT may be better for lower post-HSCT morbidity and mortality in patients with PAP, although pulmonary dysfunction can also be corrected to some extent after HSCT (73, 78).

Nichols-Vinueza et al. reported the outcome of 59 HSCT recipients with *GATA2* deficiency and compared transplant outcome based on GVHD prophylaxis (PT-CY vs. tacrolimus and methotrexate-based prophylaxis). PT-CY resulted in a significantly reduced incidence of grade III–IV acute GVHD (0 vs. 32%) and moderate-to-severe chronic GVHD (9 vs. 42%) without increase of relapse (0 vs. 5.2%) compared to that in patients who were administered tacrolimus and methotrexate-based prophylaxis (74). PT-CY may be a promising HSCT strategy in patients with *GATA2* deficiency, although the exact mechanism of deteriorating GVHD is still unclear. Hofmann et al. comparatively analyzed the transplant outcomes of pediatric patients with *GATA2* deficiency with those of patients without, and revealed that neurologic toxicities (six of 15 cases) and thrombotic events (eight out of 15 cases) were more common in patients with *GATA2* deficiency, although the underlying mechanism is uncertain (79). The risk of acute and chronic GVHD was not different based on the existence of germline *GATA2* mutation, and second malignancy was rare with a median follow up of nearly six years after HSCT.

## Transplantation outcome

Most reports about HMMs are case reports and case series, and large-scale prospective study of HSCT outcome for HMMs is lacking. Even retrospective analysis is highly limited due to its rarity (**Table 2**).

**Table 2:** Previous reports about HSCT for HMMs.

Author	Germline mutation	Patient number	Hematologic disorder	Median age at HSCT	Donor source	GVHD prophylaxis	Conditioning	Grade III–IV aGVHD	Moderate–severe cGVHD	Outcome
Grossman (73)	*GATA2*	14	MDS 12 CMML 1 EBV-LPD 1	33	MR-PB 4 UR-PB 4 UCB 4 HaploBM 2	TAC+SIR (MR/UR, UCB) PT-CY (Haplo)	Flu+TBI 2Gy (MR/UR) Flu+CY+TBI 2Gy (UCB) Flu+CY+TBI 2Gy (Haplo)	21%	NA	8/14 (57%) are alive at a median follow-up of 3.5 years (range, 12 months to 5 y)
Parta (64)	*GATA2*	22	AML 2 MDS 20	26	MR-BM 1 MR-PB 1 UR-BM 6 UR-PB 7 HaploBM 7	TAC+MTX (MR/UR) PT-CY (Haplo)	BU-based regimen (4 days)	MR/UR 26% Haplo PT-CY 0%	MR/UR 46% Haplo PT-CY 28%	2-y OS 86%
Nichols-Vinueza (74)	*GATA2*	59	AML 2 CMML 1 MDS 39 Other 17	Mean 28.4	MR/UR TAC/MTX 19 MR/UR PT-CY 23 Haplo PT-CY 17		Flu+BU (MR/UR) Flu+CY+BU+TBI 4Gy (Haplo)	TAC/MTX 32% MR/UR PT-CY 0% Haplo PT-CY 6%	TAC/MTX 42% MR/UR PT-CY 9% Haplo PT-CY 24%	TAC/MTX 4-y OS 78.9% MR/UR PT-CY 4-y OS 82.2% Haplo PT-CY 4-y OS 93.3%
Duployez (16)	*DDX41*	Mut 35 WT 288	AML	Mut 61 WT 54	NA	NA	NA	NA	NA	Mut 5-y relapse 16% WT 5-y relapse 31%
Alkhateeb (80)	*DDX41*	12	MDS/AML	NA	NA	NA	NA	NA	NA	2-y OS 87%

aGVHD, acute graft-versus-host disease; BU, busulfan; cGVHD, chronic graft-versus-host disease; CY, cyclophosphamide; Flu, fludarabine; Haplo, haploidentical; HSCT, hematopoietic stem cell transplantation; MDS, myelodysplastic syndromes; MR, matched related; MTX, methotrexate; Mut, germline mutationNA; NA, Not available; OS, overall survival; PT-CY, posttransplant-cyclophosphamide; SIR, sirolimus; TAC, tacrolimus; TBI, total body irradiation; UR, unrelated; WT, wild-type; y, year.

Since the prevalence of patients with germline *DDX41* mutations is relatively high among patients with HMMs, retrospective comparison of patients with and without germline *DDX41* mutations using large-scale data of some clinical trials was recently reported by Duployez et al.; 191 patients with AML having *DDX41* germline mutations and 1,604 *DDX41* wild-type patients with AML were compared (16). Interestingly, AML with germline *DDX41* mutation displayed a specific relapse kinetics, with a lower short-term relapse rate and higher late relapse risk. Some previous reports had suggested apparently favorable outcomes in AML with germline *DDX41* mutations (11, 51, 80); however, long-term follow up may be necessary to evaluate the genuine prognosis of AML with germline *DDX41* mutations. Regarding the role of HSCT, Duployez et al. showed that HSCT in first CR for AML with germline *DDX41* mutation may contribute to the suppression of late relapse, although OS was not significantly different (16).

Transplantation outcomes in *GATA2* deficiency have been prospectively analyzed in some clinical trials (73, 74, 78), perhaps because the unique clinical features promoted participation in clinical trials. Many of the studies on *GATA2* deficiency included tens of patients, mostly children, adolescents, or young adults. In children and adolescents, OS and disease-free survival (DFS) after HSCT in bone marrow failure, MDS, or AML in patients with *GATA2* deficiency were 65% and 51%, and those were comparable to the values in patients without *GATA2* deficiency (81). Other studies also reported good survival rate after HSCT; four- or five-year OS was 78–93% (64, 74). However, reported HSCT outcomes for *GATA2* deficiency should be interpreted with caution, since the proportion of MDS with high-risk features, such as monosomy 7, and severity of concomitant organ dysfunction can affect the outcomes.

Transplant outcomes for FPD-MM, AML with germline *CEBPA* mutations, and other HMMs have not been reported yet, and HSCT outcomes of patients with sporadic relevant mutations still need to be extrapolated. Waidhauser et al. analyzed 674 patients with AML, who underwent HSCT in first CR (*RUNX1* mutation; positive 183, negative 491). Two-year overall survival (OS) was not significantly different between *RUNX1* mutation-positive and -negative (67.7 vs 66.1%, p = 0.7). Considering the poor prognosis of AML with *RUNX1* mutation (33), HSCT in first CR was considered to overcome the unfavorability. In patients with AML having somatic biallelic *CEBPA* mutations and having underwent HSCT, five-year OS was 71.8%, which was not significantly different from that in patients with AML having somatic biallelic *CEBPA* mutations and receiving consolidation chemotherapy alone (82).

## Concluding remarks

We summarized the concurrent available reports about HSCT in patients with HMMs. Although HMMs have recently been noticed more widely, there is too little evidence about recipients as well as donors. Increasing awareness of HMMs would ensure better management by physicians, which could surely improve patient outcomes.

## Author contributions

TT wrote the manuscript. HH and YH critically revised the paper and approved the final version. ND supervised the study. All authors contributed to the article and approved the submitted version.

## Conflict of interest

The authors declare that the research was conducted in the absence of any commercial or financial relationships that could be construed as a potential conflict of interest.

## Publisher’s note

All claims expressed in this article are solely those of the authors and do not necessarily represent those of their affiliated organizations, or those of the publisher, the editors and the reviewers. Any product that may be evaluated in this article, or claim that may be made by its manufacturer, is not guaranteed or endorsed by the publisher.
